# Impacts of simulated erosion and soil amendments on greenhouse gas fluxes and maize yield in Miamian soil of central Ohio

**DOI:** 10.1038/s41598-017-18922-6

**Published:** 2018-01-11

**Authors:** Yanru Liang, Rattan Lal, Shengli Guo, Ruiqiang Liu, Yaxian Hu

**Affiliations:** 1 College of Natural Resources and Environment, Northwest A&F University, Yangling, 712100 Shaanxi P.R. China; 2 Carbon Management and Sequestration Center, School of Environment and Natural Resources, The Ohio State University, Columbus, 43210 OH USA; 3 Institute of Soil and Water Conservation, Northwest A&F University, Yangling, 712100 Shaanxi P.R. China

## Abstract

Erosion-induced topsoil loss is a threat to sustainable productivity. Topsoil removal from, or added to, the existing surface is an efficient technique to simulate on-site soil erosion and deposition. A 15-year simulated erosion was conducted at Waterman Farm of Ohio State University to assess impacts of topsoil depth on greenhouse gas (GHG) emissions and maize yield. Three topsoil treatments were investigated: 20 cm topsoil removal, 20 cm topsoil addition, and undisturbed control. Results show that the average global warming potential (GWP) (Mg CO_2_ Eq ha^−1^ growing season^−1^) from the topsoil removal plot (18.07) exhibited roughly the same value as that from the undisturbed control plot (18.11), but declined evidently from the topsoil addition plot (10.58). Maize yield decreased by 51% at the topsoil removal plot, while increased by 47% at the topsoil addition plot, when compared with the undisturbed control (7.45 Mg ha^−1^). The average GWP of erosion-deposition process was 21% lower than that of the undisturbed control, but that greenhouse gas intensity (GHGI) was 22% higher due to lower yields from the topsoil removal plot. Organic manure application enhanced GWP by 15%, and promoted maize yield by 18%, but brought a small reduction GHGI (3%) against the N-fertilizer application.

## Introduction

In agricultural ecosystem, sustainable food production and mitigation of greenhouse gas (GHG) emissions have been concerned by agricultural or environmental scientists, especially under future climate conditions. Accelerated erosion is one of the most prevalent forms of soil degradation in the world^1,2^, which poses major threat to food security^3^ and a significant impact on GHG emissions^4^. Erosion translocate sediment and soil organic C laterally across landscapes (0.5–0.6 Pg C year^−1^)^5^, potentially causing approximately 0.8–1.2 Gt C year^−1^ emissions into the atmosphere, while burying 0.4–0.6 Gt C year^−1^ by deposition processes^2^. Although erosion-induced GHG emissions are dominated by CO_2_, the fluxes of CH_4_ and N_2_O are also considerable^6^. IPCC (Intergovernmental Panel on Climate Change) (2007) stated^7^ that the emissions of N_2_O and CH_4_ have global warming potentials (GWPs) of 310 and 21 times that of CO_2_, which have not yet been adequately investigated. Soil erosion comprises three stages: detachment, transport/redistribution, and deposition^8^. The first two stages, detachment and transportation, lead to increased mineralization and emission of CO_2_. However, the prevalence of anaerobic conditions at the depositional stage reduces the emission of CO_2_ and leads to flux of CH_4_ and N_2_O^9^. Up to now, there is no systematically assessment on grain yield and GHG emissions under soil erosion-deposition events.

In fact, it is difficult to detect the decline of productivity that results from erosion directly, because the productivity reduction caused by erosion often occurs so slowly that it may not be recognized until crop production is no longer economically viable^10^. Moreover, improved technology often masks productivity decline caused by erosion, leading to increased rather than decreased yields^4,10^. Various indirect methods (e.g., the comparative-plot method, transect method, and desurfacing experiments) have been carried out extensively in the study of erosion-productivity relationships^10^. The simplest method is to artificially remove topsoil (which is also referred to as desurfacing experiments^10,11^). Previous studies have reported that the yield reduction rate was faster after the top 40 cm soil was eroded, but became slower if the deeper soil was lost^12^. Moreover, desurfacing approach can also help eliminate the inherent variability of topsoil depth and landscape position^13^.

Restoration of degraded soils is a high priority in global scale^4^. When soil carbon pool in degraded cropland increased by one ton, crop yield would increase by 20–40 kg ha^−1^ for wheat, 10–20 kg ha^−1^ for maize, and 0.5–1 kg ha^−1^ for cowpeas^2^. Substantial studies have reported the restored productivity of de-surfaced soils by amending with fertilizer or manure^14–16^.

In this study, we investigated the impacts of simulated soil erosion-deposition (after 15 years of establishment) on greenhouse gas (GHG; CO_2_, N_2_O, and CH_4_) emissions and maize yield, via topsoil depth (TSD) removal and addition^8,17^ treatments during the growing season, under N-fertilizer and organic manure amendments with no-till management.

## Results

### Soil temperature, moisture and GHG emissions

**Figure 1:**
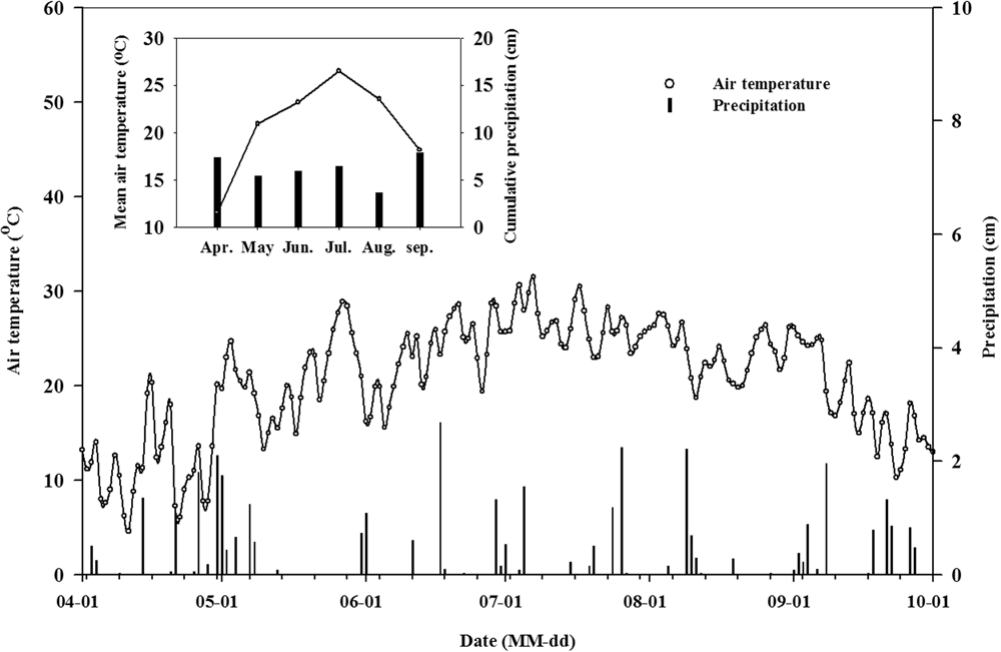
Daily temperature and precipitation distribution in 2012; the inset shows the cumulative monthly precipitation and the mean monthly temperature.

Figure 1 displays the diurnal air temperature and precipitation distribution in 2012; the inset shows the cumulative monthly precipitation and mean monthly temperature in 2012 (OARDC). Mean air temperature showed an increase from April (11.55 °C) to July (26.53 °C), followed by a decrease thereafter. Monthly cumulative precipitation varied between 3.69 and 7.96 cm from April to September, with the highest precipitation in September and lowest precipitation in August.

**Figure 2:**
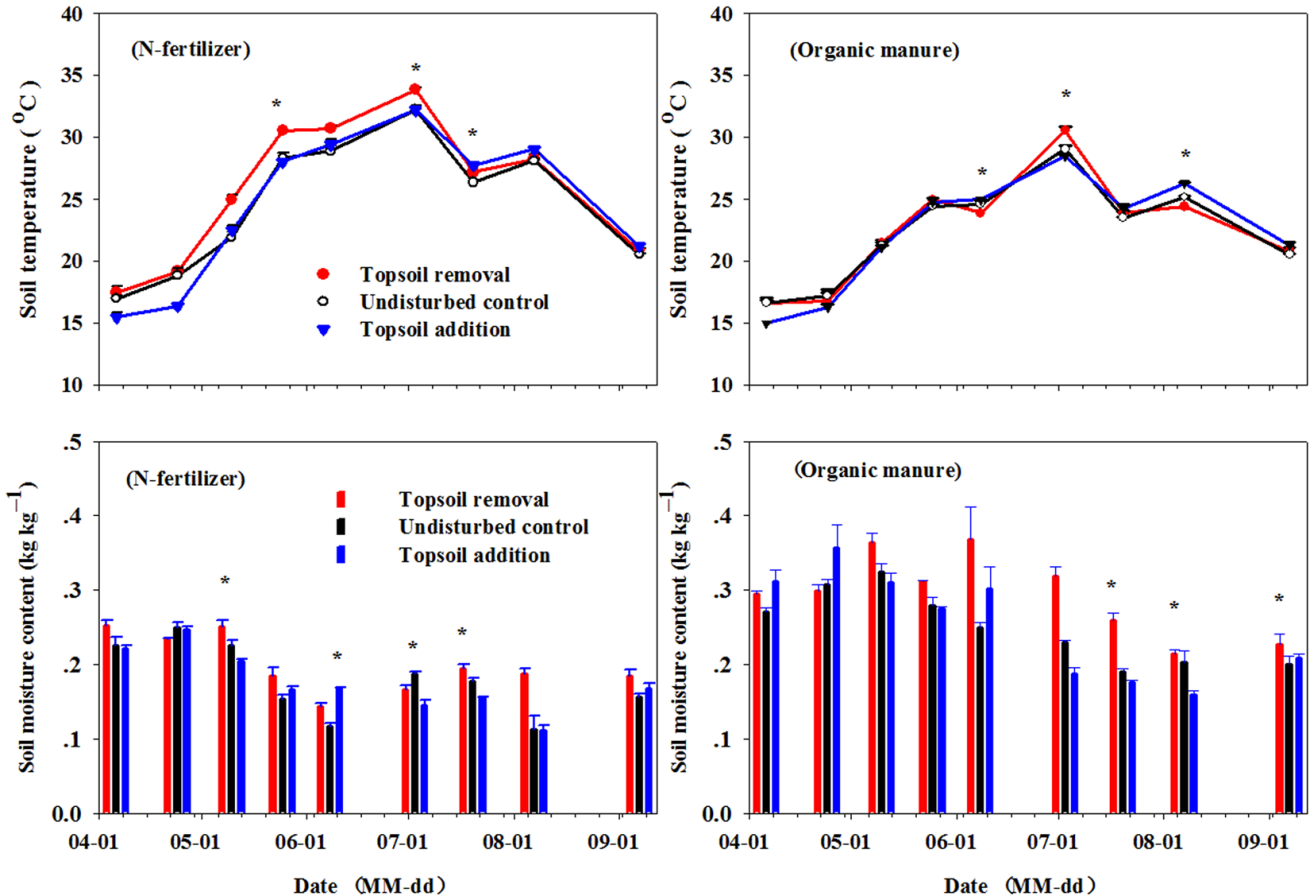
Soil temperature and soil moisture content affected by simulated erosion under N-fertilizer and organic manure application during the growing season in 2012. The error bar represent the standard error (n = 3). The asterisk (^*^) represents statistical significance within a sampling date at p ≤ 0.05.

Figure 2 shows the variation of soil temperature and soil moisture content at 0–10 cm impacted by simulated erosion under N-fertilizer and organic manure application during the growing season in 2012. Like air temperature, soil temperature showed an increase from April to July, followed by a decrease thereafter. During the growing season, mean soil temperatures for the three TSD treatments were 25.65, 24.75, and 24.92 °C respectively for top soil removal, undisturbed control, and topsoil addition. Obviously higher soil temperature trend was observed for topsoil removal under N-fertilizer application. While, mean soil moisture content for topsoil removal was significantly higher than other two TSD treatments (*P* = 0.0372) at 10 cm depth.

**Figure 3:**
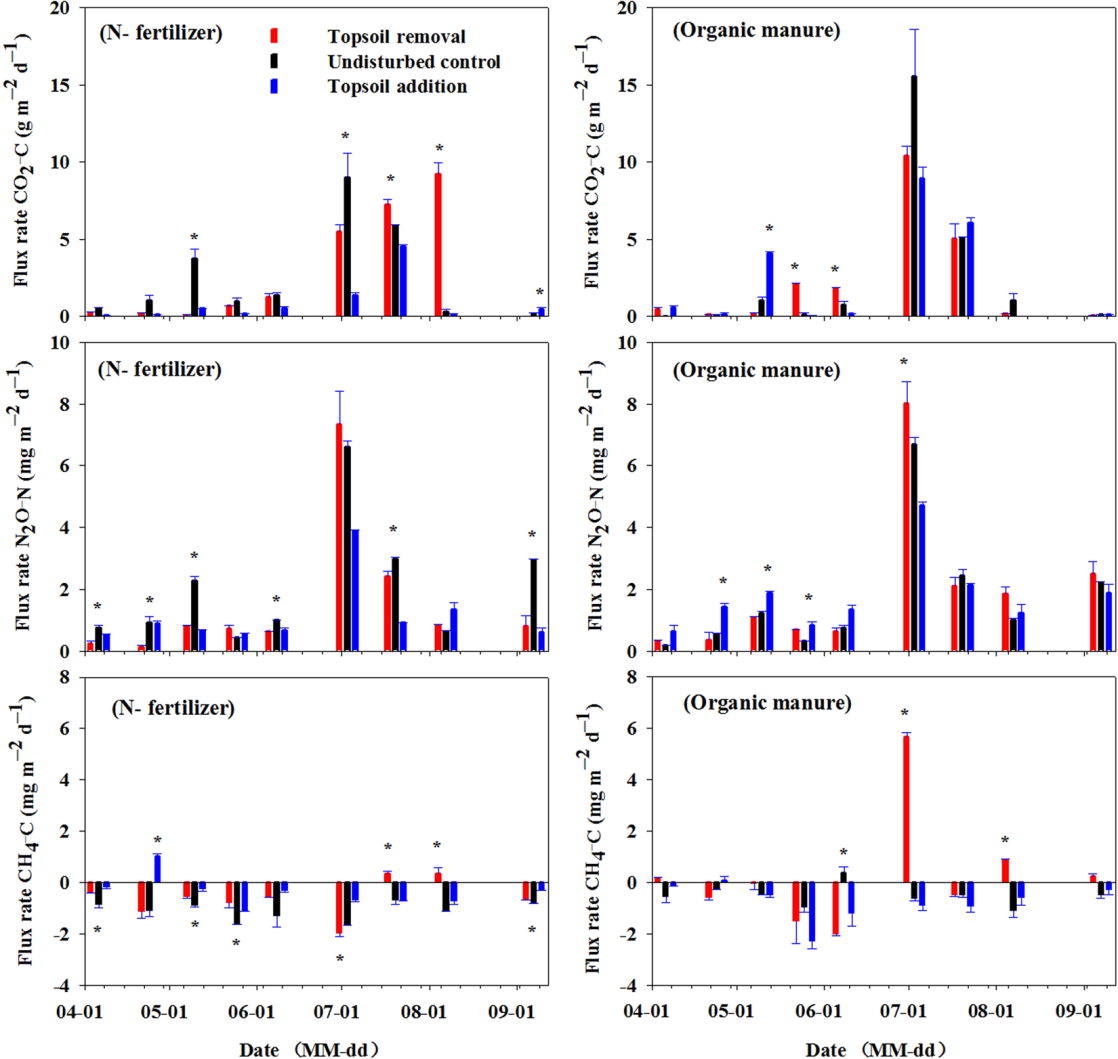
Daily CO_2_, N_2_O and CH_4_ fluxes affected by simulated erosion under N-fertilizer and organic manure application during the growing season in 2012. The error bar represent the standard error (n = 3). The asterisk (^*^) represents statistical significance within a sampling date at p ≤ 0.05.

Figure 3 shows the effects of simulated erosion on CO_2_ fluxes under N-fertilizer and organic manure application during the growing season in 2012. All three TSD treatments were persistent CO_2_ sources both for N-fertilizer and organic manure application during the study. Soil CO_2_ fluxes differed among seasons, which stirred by fertilizer application and reached the maxima at the peak of air temperature and corn growth^18^. Under N-fertilizer application, CO_2_ fluxes in all three TSD treatments were below 4 g C m^−2^ d^−1^ from April 6^th^ to June 8^th^, 2012. CO_2_ fluxes increased sharply from July to August, and observed the maximum fluxes (g C m^−2^ d^−1^) on August 7^th^ (9.22), July 3^rd^ (8.98) and July 20^th^ (4.55) respectively for topsoil removal, undisturbed control and topsoil addition. For organic manure application, peak CO_2_ fluxes (g C m^−2^ d^−1^) appeared on July 3^rd^, which were 10.4, 15.54 and 8.94 respectively for topsoil removal, undisturbed control and topsoil addition. For N-fertilizer application, there was a 19% increase in the topsoil removal treatment and a 67% decrease in the topsoil addition treatment compared with undisturbed control (Table 1). For soil receiving organic manure, no significant difference observed among the three TSD treatments (Table 1). Average cumulative CO_2_ emissions for the entire growing season significantly differed among the three TSD treatments (*P* = 0.0003) (Table 2).

**Table 1:** Effects of simulated erosion on cumulative greenhouse gas (GHG) emissions, global warming potential (GWP), greenhouse gas intensity (GHGI) under two fertilizer application during a growing season.

Treatment	CO_2_–C (Mg ha^−1^)	N_2_O–N (kg ha^−1^)	CH_4_–C (kg ha^−1^)	GWP	GHGI
**N-fertilizer**					
Topsoil removal	**5.11 ± 0.03a**	**2.73 ± 0.09b**	**−0.92 ± 0.00b**	**20.04**	**5.12**
Undisturbed control	**4.30 ± 0.06b**	**3.38 ± 0.01a**	**−1.78 ± 0.01a**	**17.36**	**2.40**
Topsoil addition	**1.44 ± 0.02c**	**1.95 ± 0.01c**	**−0.58 ± 0.04c**	**6.21**	**0.69**
**Organic manure**					
Topsoil removal	**3.98 ± 0.01**	**3.41 ± 0.01a**	**0.57 ± 0.13a**	**16.27**	**4.98**
Undisturbed control	**4.80 ± 0.42**	**2.94 ± 0.00b**	**−0.77 ± 0.13b**	**19.01**	**2.48**
Topsoil addition	3.72 ± 0.04	2.98 ± 0.00b	−1.24 ± 0.18b	15.06	1.16

Values are given as mean ± s.e.m. (n = 3). Values in each column followed by different letters are statistically different at *p* ≤ 0.05.

GWP (Mg CO_2_ Eq ha^−1^ growing season^−1^) = CH_4_ × 21 + N_2_O × 310 + CO_2_

GHGI (Mg CO_2_ Eq Mg grain yield growing season^−1^) = GWP/grain yield

**Table 2:** Statistical significance of fertilizer, topsoil depth (TSD) and fertilizer × TSD interaction effects on crop yield, aboveground residue and cumulative greenhouse gas (GHG) emissions in 2012 growing season.

	*P* > *F*				
	Grain yield	Above ground residue	CO_2_	N_2_O	CH_4_
Fertilizer	NS	0.0151	0.0345	<0.0001	<0.0001
Topsoil depth	<0.0001	0.0012	0.0003	<0.0001	<0.0001
Fertilizer × TSD	0.0256	NS	0.0014	<0.0001	<0.0001

Figure 3 shows the effects of simulated erosion on N_2_O fluxes under N-fertilizer and organic manure application during the growing season in 2012. Three TSD treatments were also persistent sources of N_2_O fluxes both for receiving N-fertilizer and organic manure during the study. Soil N_2_O fluxes also displayed a small increase on May 10^th^ after the fertilizer application, and a peak flux coincided with the peak air and soil temperatures in July. The results indicate that the CO_2_ and N_2_O fluxes were stimulated by fertilizer application and soil temperature rise. N_2_O fluxes fluctuated below 3 mg N m^−2^ d^−1^ from April to June, and rose rapidly from June 8^th^, got the maxima fluxes on July 3^rd^ both under N-fertilizer application (7.35, 6.59 and 3.90 mg N m^−2^ d^−1^ respectively for topsoil removal, undisturbed control and topsoil addition) and organic manure application (8.02, 6.70 and 4.71 mg N m^−2^ d^−1^ respectively for topsoil removal, undisturbed control and topsoil addition), then declined to the original level (<3 mg N m^−2^ d^−1^) thereafter until September (Fig. 3). Our result agree with the statement that the N_2_O emission often characterized by a short time of very high flux rates that make up a substantial part of the total annual loss^19^. Under N-fertilizer application, cumulative N_2_O emission decreased by 19% and 42% respectively for topsoil removal and topsoil addition treatments, compared with the undisturbed control (Table 1). For organic manure application, cumulative N_2_O emission increased by 16% for topsoil removal compared with the undisturbed control, and no significant difference was observed between the undisturbed control and topsoil addition (Table 1). Average cumulative N_2_O emissions for the entire growing season significantly differed among the three TSD treatments (*P* < 0.0001) (Table 2).

Figure 3 shows the effects of simulated erosion on CH_4_ fluxes under N-fertilizer and organic manure application during the growing season in 2012. Under N-fertilizer application, CH_4_ fluxes in the three TSD treatments were generally low (i.e., averaged <2 mg C m^−2^ d^−1^) and broadly taken up during the growing season. While, under organic manure application, CH_4_ fluxes fluctuated widely, and a peak positive CH_4_ flux (5.68 mg C m^−2^ d^−1^) observed on July 3^rd^ for topsoil removal. For N-fertilizer application, all three TSD treatments were net CH_4_ sinks from the atmosphere during the growing season (Table 1). Cumulative CH_4_ uptake decreased for both topsoil removal (by 49%) and topsoil addition (by 67%) compared with the undisturbed control (−1.78 kg C ha^−1^ growing season^−1^) (Table 1). Under organic manure application, topsoil removal was net CH_4_ source, and two other TSD treatments were net CH_4_ sinks during the growing season, cumulative CH_4_ emission increased by 174% for topsoil removal compared with the undisturbed control (−0.77 kg C ha^−1^ growing season^−1^) (Table 1). Average cumulative CH_4_ emissions significantly differed among the three TSD treatments (p < 0.0001) for the entire growing season (Table 2).

Soil temperatures were negatively correlated with soil moisture contents both under N-fertilizer application (*P* = 0.0232, 0.0397 and 0.0046 respectively for topsoil removal, undisturbed control and topsoil addition), and organic manure application (*P* = 0.0350 for topsoil addition). Soil temperature was positively correlated with CO_2_ fluxes (*P* = 0.0787) and N_2_O fluxes (*P* = 0.0918) from the topsoil removed plot under N- fertilizer application. Similar and much stronger correlations were also found between soil temperature and CO_2_ fluxes (*P* = 0.0138 and 0.0453 respectively for topsoil removal and undisturbed control) as well with N_2_O fluxes (*P* = 0.0194 and 0.0615 respectively for topsoil removal and undisturbed control) under organic manure application. CO_2_ fluxes were positively correlated with N_2_O flux both under N-fertilizer (*P* = 0.0073, 0.0002 and 0.0024 respectively for topsoil removal, topsoil addition and undisturbed control) and organic manure application *P* = 0.0025 and 0.0023 respectively for topsoil removal and topsoil addition; *P* < 0.0001 for undisturbed control). The CH_4_ fluxes were negatively correlated with soil temperature both under N-fertilizer application (*P* = 0.0466 for undisturbed, *P* = 0.0255 for topsoil addition) and organic manure application (*P* = 0.0734 for topsoil addition).

### Soil bulk density and SOC, total N content

**Figure 4:**
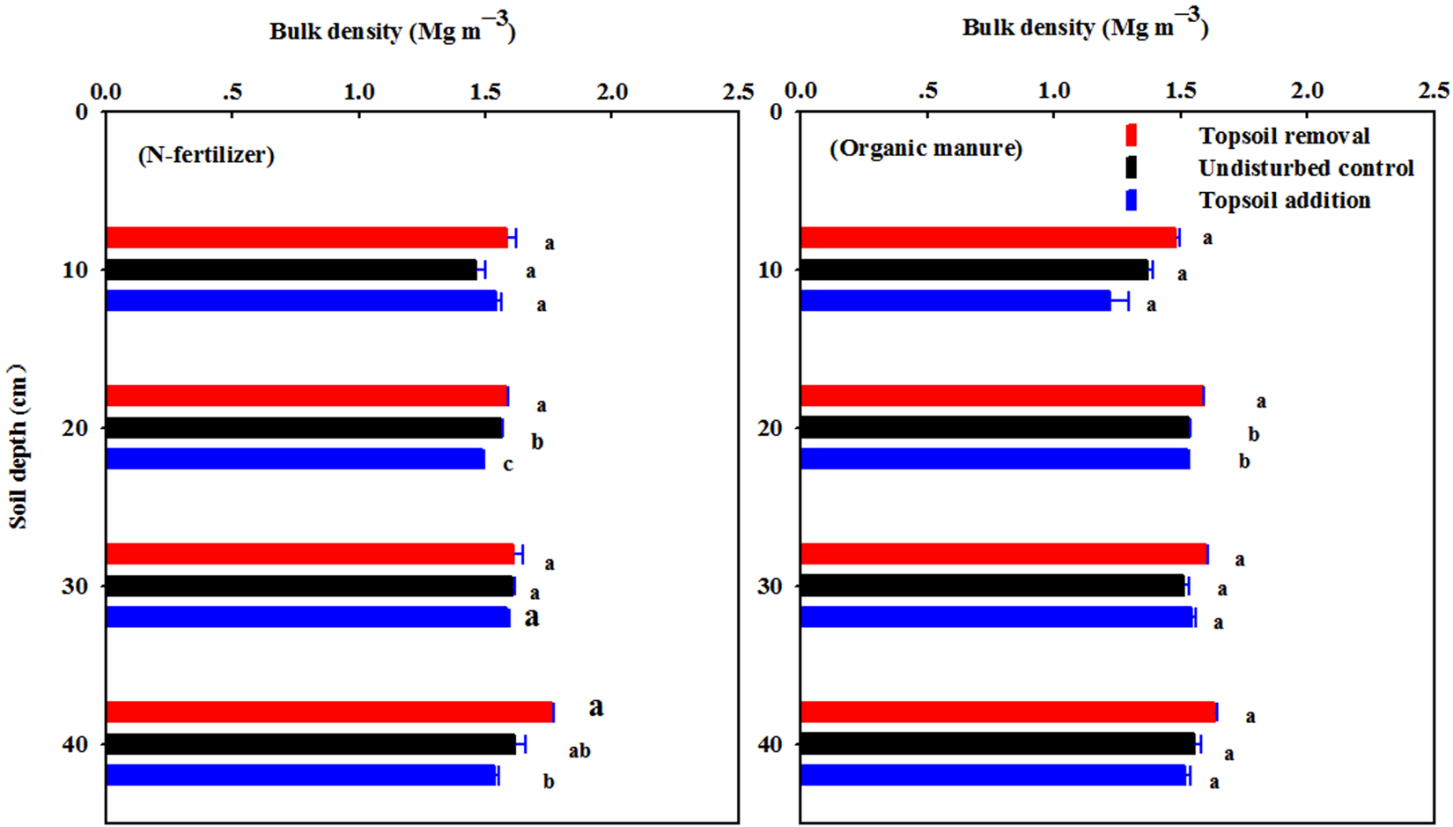
Soil bulk density affected by simulated erosion under N-fertilizer and organic manure application. The error bars represent the standard error (n = 3). Values in each soil depth followed by different letters are statistically different at p ≤ 0.05.

Figure 4 shows the soil bulk density affected by simulated soil erosion under N-fertilizer and organic manure application. Soil bulk density was higher for topsoil removal and lower for topsoil addition at every soil layer depth from 0–40 cm, significant difference were observed among the three TSD treatments both under N-fertilizer (at 20 and 40 cm soil layer depth), and organic manure (20 cm soil layer depth) application. Soil bulk density for soil receiving organic manure was lower than soil with N-fertilizer application at every soil layer depth from 0–40 cm, with significant difference observed at 0–10 cm soil layer depth.

**Figure 5:**
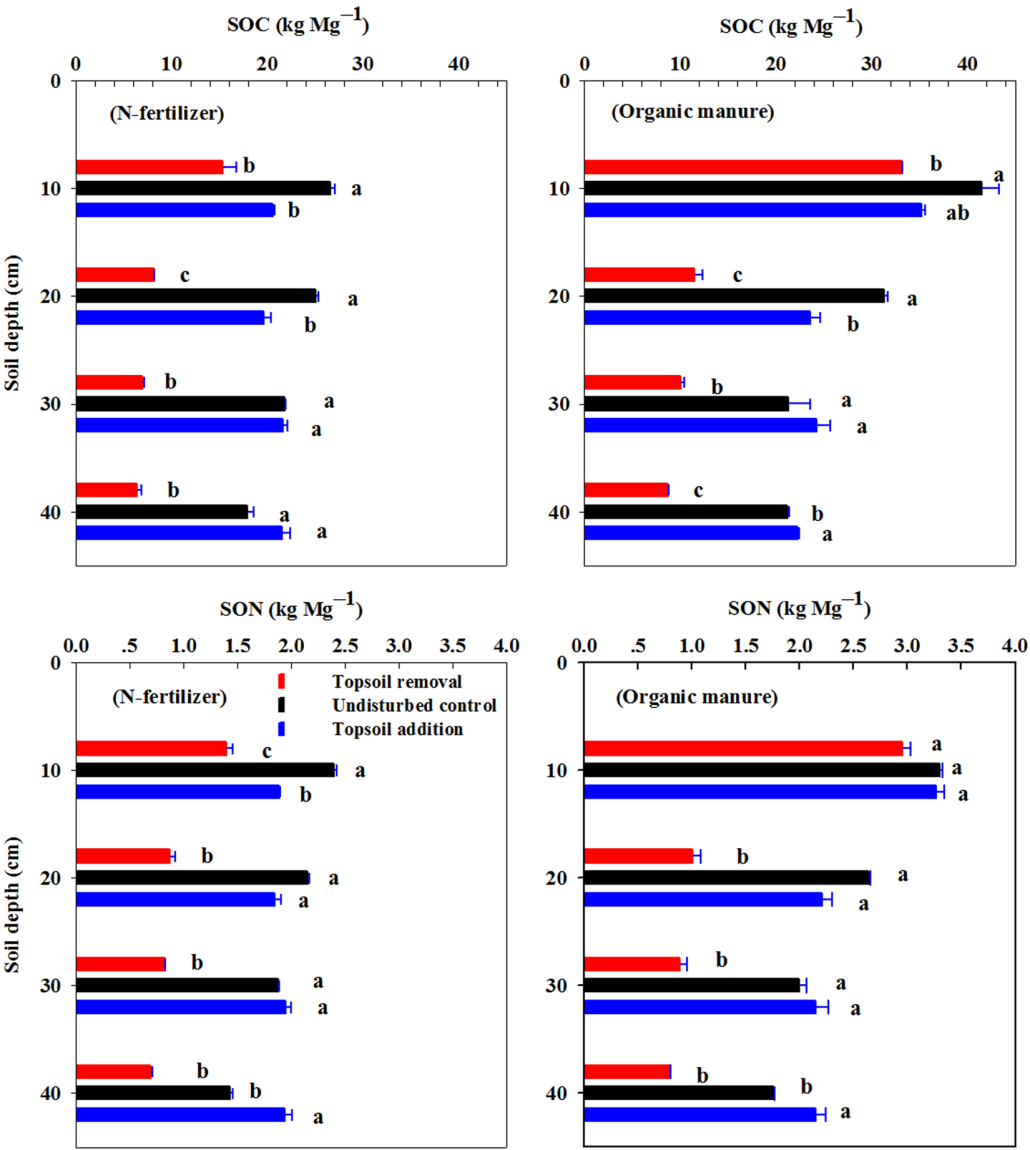
SOC and SON affected by simulated erosion under N-fertilizer and organic manure application. The error bars represent the standard error (n = 3). Values in each soil depth followed by different letters are statistically different at p ≤ 0.05.

Figure 5 shows the SOC and total N content affected by simulated erosion under N-fertilizer and organic manure application. Significant differences of SOC and total N at the top soil layers displayed inverse patterns from that at the lower layer. At the soil layer of 0–10 and 10–20 cm, the SOC and total N were in the order of undisturbed control > topsoil addition > topsoil removal, while in reverse order of topsoil addition > undisturbed control > topsoil removal at 30–40 cm soil layer depth. For topsoil addition, the migrated topsoil might lose part of C from decomposition due to soil disturbance^20,21^; meanwhile, the former topsoil buried under the plough depth was preserved from decomposition and mineralization^22–24^. Soil receiving organic manure had higher SOC and total N content than soil with N-fertilizer application at every soil layer depth from 0–40 cm.

### Soil GWP, GHGI and maize yield

The GWP (Table 1) increased by 16% for topsoil removal and decreased by 64% for topsoil addition compared with the undisturbed control (17.36 Mg CO_2_ Eq ha^−1^ growing season^−1^) under N-fertilizer application; and GWP decreased by 14% and 21% respectively for topsoil removal and topsoil addition compared with the undisturbed control (19.01 Mg CO_2_ Eq ha^−1^ growing season^−1^) under organic manure application. The mean GWP for topsoil removal and topsoil addition decreased by 24% under N-fertilizer application, and decreased by 18% under organic manure application, compared with the undisturbed control.

For N-fertilizer application, the GHGI (Table 1) increased by 113% for topsoil removal and decreased by 71% for topsoil addition, compared with the undisturbed control (2.40 Mg CO_2_ Eq Mg^−1^ grain yield growing season^−1^); the average GHGI of the topsoil removal and topsoil addition was 21% higher than that of the undisturbed control. Under organic manure application, the GHGI increased by 101% for topsoil removal and decreased by 53% for topsoil addition, compared with the undisturbed control (2.48 Mg CO_2_ Eq Mg^−1^ grain yield growing season^−1^); the average GHGI of the topsoil removal and topsoil addition was 24% higher than that of the undisturbed control (Table 1).

**Figure 6:**
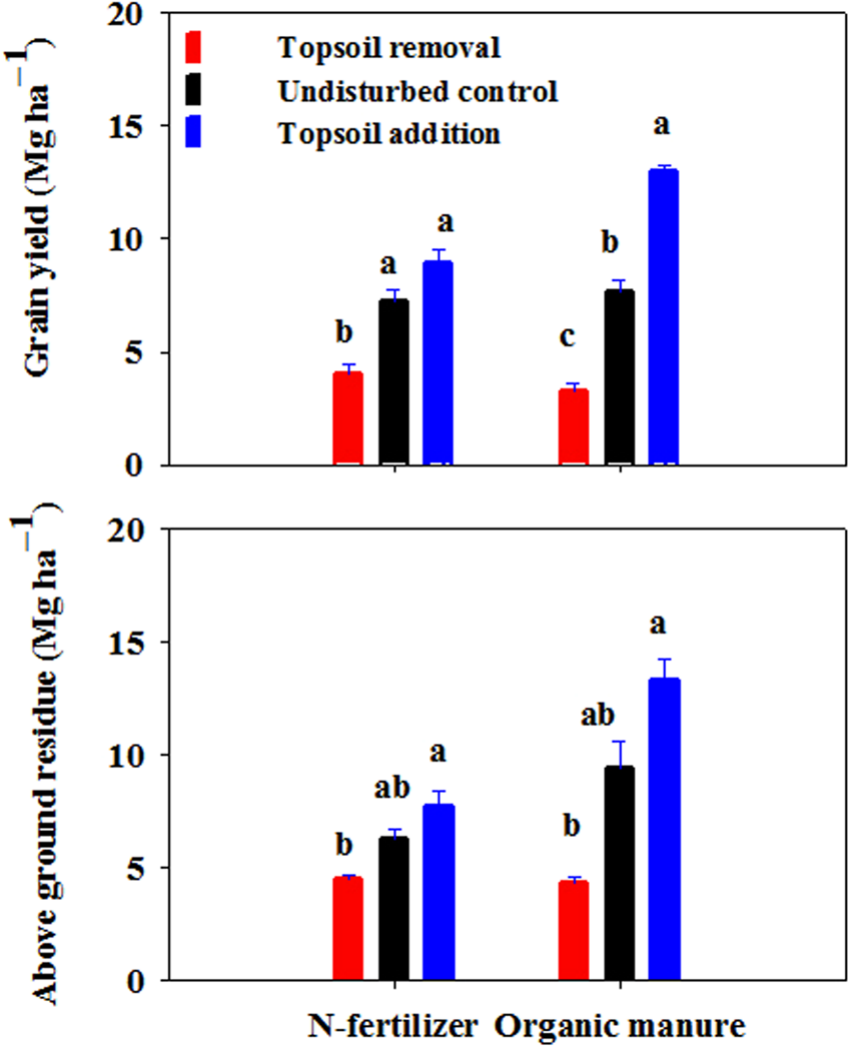
Grain yield and above ground residue affected by simulated erosion under N-fertilizer and organic manure application in 2012. The error bars represent the standard error (n = 3). Values in each soil depth followed by different letters are statistically different at p ≤ 0.05.

Figure 6 shows the grain yield and above ground residue affected by simulated erosion under N-fertilizer and organic manure application in 2012. The grain yield significantly decreased (by 45% under N-fertilizer, by 57% under organic manure) for topsoil removal, while increased (by 24% under N-fertilizer, by 69% under organic manure) for topsoil addition compared with the undisturbed control (7.23 Mg ha^−1^ under N-fertilizer, 7.67 Mg ha^−1^ under organic manure). The average grain yield of topsoil removal and topsoil addition decreased by 10% under N-fertilizer application, and increased by 6% under organic manure application, compared with the undisturbed control (7.23 Mg ha^−1^ under N-fertilizer, 7.67 Mg ha^−1^ under organic manure).

The above ground residue decreased (by 28% under N-fertilizer, by 54% under organic manure) for topsoil removal, and increased (by 23% under N-fertilizer, by 41% under organic manure) for topsoil addition, compared with the undisturbed control (6.31 Mg ha^−1^ under N-fertilizer, 9.46 Mg ha^−1^ under organic manure. The average above ground residue of topsoil removal and topsoil addition decreased by 2% under N-fertilizer application, and decreased by 7% under organic manure application compared with the undisturbed control (6.31 Mg ha^−1^ under N-fertilizer, 9.46 Mg ha^−1^ under organic manure). Significant difference were observed among three TSD treatments both for grain yield (*P* < 0.001) and above ground residue (*P* = 0.0256) (Table 2).

## Discussion

Topsoil remove and addition can not only significantly affect the greenhouse gases emissions, but also changes the crop yield (Table 1 and Fig. 6), which indicated that the erosion-deposition process can significantly alter the global warming effects, soil nutrient status and soil productivity during the erosion events.

The average cumulative CO_2_ emission in our study was 3.89 Mg C ha^−1^ growing season^−1^; the value fell into the range of seasonal CO_2_ emission reported by several global studies^20,25–27^. In the present study, cumulative CO_2_ emission significantly increased following 20 cm topsoil removal and decreased following 20 cm topsoil addition throughout the maize growing season compared with the undisturbed soil under N-fertilizer application (Table 1). The difference of cumulative CO_2_ emissions among three TSD treatments could be explained by the soil temperature variation that caused by aboveground coverage shade^28^. Soil temperature was the primary drive to CO_2_ flux^21,28–31^. The enhanced cumulative CO_2_ emission at the eroded site may primarily result from its higher soil temperature of 25.9 °C (from 17.5 to 33.9 °C) with less aboveground coverage shade. The reduced cumulative CO_2_ emission at the depositional site probably due to its lower soil temperature of 24.7 °C (from 15.5 to 32.2 °C) owing to its dense above ground coverage shade (Figs 2 and 6). In addition, the reduced cumulative CO_2_ emissions at depositional site probably also caused by their lower substrate availability (e.g., SOC)^28^ in surface soil (Table 1 and Fig. 5). While, under organic manure application, no significant difference observed among the three TSD treatments for cumulative CO_2_ emissions (Table 1), which probably due to their similar average soil temperatures (respectively 22.6, 22.5 and 22.5 °C) (Fig. 2). It was worthwhile to note that the cumulative CO_2_ emission for the organic manure applied plot (4.16 Mg C ha^−1^ growing season^−1^) was much greater than that from the plot receiving N-fertilizer (3.62 Mg C ha^−1^ growing season^−1^) (*P* = 0.0345). This was probably due to the greater carbon substrate (SOC) under organic manure application^20^ (Fig. 5). Soil moisture content did not respond to the cumulative CO_2_ emission in our study, probably because the soil seldom underwent prolonged drought^28^. Similar results also reported by Sheng *et al*. (2010). Smith *et al*. reported that the release of CO_2_ by aerobic respiration is primarily driven by soil temperature, but becomes moisture-dependent as soil dries out^32^.

Among the three TSD treatments, undisturbed plot exhibited the largest cumulative N_2_O emission under N-fertilizer application. This can possibly attribute to the greater SOC and total N content in 0–20 cm soil layer depth (Table 2 and Fig. 5), as N_2_O emission depended on SOC and total nitrogen contents, bulk density, clay fraction and soil moisture content that regulates N_2_O production via microbial de-nitrification as well as nitrification^33^. The second largest cumulative N_2_O emission was at the eroded site under N-fertilizer, which might due to its higher soil temperature^32^ and greater soil bulk density (Table 2, Figs 2 and 4). The results are in good line with previous studies conducted with an intensively farmed organic soil in North Central Ohio, which reported that N_2_O flux was positively related to soil temperature and CO_2_ flux^34^. The least cumulative N_2_O emission at the depositional site may be determined by its lower soil temperature and lower SOC, total N content (Table 2, Figs 2 and 5). In addition, the eroded site displayed the highest cumulative N_2_O emission under organic manure application, probably ascribed to the anaerobic conditions^35^ in soil pore space caused by higher soil moisture content and greater soil bulk density (Figs 2 and 4). The result is in agreement with a previous report that N_2_O emissions as a result of denitrification occurred in compaction treatment^36^. Flessa *et al*., reported that total N_2_O emission from the potato field during the growing season was 2.0 (kg N ha^−1^ growing season^−1^) in 1998^19^, and Kumar *et al*., resulted 2.89 (kg N ha^−1^ year^−1^) of N_2_O emission from corn–corn cropping rotation in an Alfisol of Ohio in 2012^26^, which were similar to our results of average cumulative N_2_O emission (2.9 kg N ha^−1^ growing season^−1^) in corn field in 2012.

In this study, cumulative CH_4_ emission was positive at the eroded site under organic manure application, which was probably due to the hypoxic conditions^35^ that may have been caused by the relatively greater soil moisture (Fig. 2). Cumulative CH_4_ emissions from other TSD treatments were negative, which indicated the net CH_4_ sink during the growing season (Table 1). The low values and net uptake of CH_4_ reported in our study were consistent with those reported for cultivated soils^25,37^. Toma *et al*., (2011) reported that CH_4_ fluxes in grassland soils were −1.07 (kg C ha^−1^ growing season^−1^) in 2008 and −1.62 (kg C ha^−1^ growing season^−1^) in 2009^35^, which was similar to our results of average cumulative CH_4_ emission (−0.79 kg C ha^−1^ growing season^−1^) in agricultural soils in 2012. Despite the very low N_2_O flux from, and CH_4_ adsorption by, the corn land in our study, the flux of N_2_O and CH_4_, as two major greenhouse gases, should be studied further.

In our study, the average GWP at the eroded site (18.07 Mg CO_2_ Eq ha^−1^ growing season^−1^) generated roughly the same value with the undisturbed control (18.11 Mg CO_2_ Eq ha^−1^ growing season^−1^), but declined dramatically at the depositional site (10.58 Mg CO_2_ Eq ha^−1^ growing season^−1^). The cumulative GHG emission at the eroded site might be stimulated via the higher soil temperature caused by its less aboveground coverage shade, but limited by its lower available C substrate. The cumulative GHG emission at the depositional site declined because of its lower soil temperature and lower available C substrate in surface. In addition, the cumulative GHG emission may also be influenced by soil bulk density and soil moisture content. The results displayed that eroded site can neither play a net sink nor net source of greenhouse gases emission, while depositional site can be a net sink of GHG emission.

It is critical to consider the overall (net) effect of the erosion and deposition processes in comparison with undisturbed soil (the control). Therefore, in this paper, we compared important parameters for undisturbed control with the average values observed for the combination of the topsoil removal and topsoil addition conditions. The average value of GWP of eroded and depositional site was 21% lower than that of the undisturbed control, indicated the erosion- deposition process could be net sink of GHG emission.

Average GWP for soil receiving organic manure was increased by 15%, compared with that with N-fertilizer application. This might be due to the greater SOC and total N content and the higher soil moisture content after organic manure application (Figs 2 and 5).

Given the limited accessibility to the experimental field, gas fluxes were only collected once every 2 weeks. There might be other peaks in GHG fluxes that were not captured during our sampling regime. More frequent sampling intervals are highly recommended in the further study.

In our study, the average maize yield significantly decreased by 51% at eroded site, and significantly increased by 47% at depositional site compared with the undisturbed control (7.45 Mg ha^−1^) (*P* < 0.0001), which was coincide with maize yields on two Alfisols in central Ohio^38^, and consistent with the finding that the corn yield declined by nearly half (46%) on removal of 20 cm of the topsoil in an eroded farmland of Chinese Mollisols^16^. Crop yield usually adversely affected by the impedance of root growth, water and nutrient deficits, high bulk density, penetrometer resistance, and low field moisture capacity under erosion^10,11,16^. On the one hand, topsoil removal significantly declined the SOC and total N content, increased the soil bulk density, and further reduced the maize yield at the eroded site. On the other hand, the topsoil addition buried the former topsoil under plough depth, preserved SOC from decomposition and mineralization^22–24,39^, promoted deep root growth, and consequently resulted to greater crop productivity (Figs 4, 5 and 6). This result agree with the findings that the impacts of erosion on agricultural land are usually negative for eroded sites and may be positive for depositional sites^40,41^.

The average value of maize yield of eroded and depositional site declined by 2% compared with the undisturbed control (7.45 Mg ha^−1^), which indicate the erosion-deposition process resulted the equivalent production with the uneroded site.

Maize yield at the organic manure applied plot (7.97 Mg ha^−1^) increased by 18% compared with that from the plot receiving N-fertilizer (6.74 Mg ha^−1^). This was probably caused by the improved SOC, total N content and soil moisture content with organic manure application (Figs 2 and 5).

The average value of GHGI was 4.96, 2.43 and 0.96 Mg CO_2_ Eq Mg^−1^ grain yield growing season^−1^, respectively for eroded site, undisturbed control and depositional site. The eroded site enhanced the GHGI because of its lower grain yield, while the depositional site declined the GHGI due to its lower GWP and higher grain yield. The average GHGI of erosion-depositional site increased by 22% compared to the undisturbed control. The average GHGI for soil receiving organic manure exhibited a small reduction of 3% compared with that of N-fertilizer application.

In summary, our results displayed that eroded site can neither play a net sink nor net source of greenhouse gases emission, while depositional site can be a net sink of GHG emission. Eroded site significantly reduced maize yield, while depositional site significantly enhanced the maize yield. The erosion-deposition process declined the GWP, not changed maize yield, and increased the GHGI. Soil with organic manure application enhanced GWP, improved maize yield and slightly reduced GHGI compared with soil receiving N-fertilizer.

It was worthwhile to note that our results were merely concluded based on the assumption that the area of erosion equals the area of deposition. However, in natural field, eros ion tends to be dissipated over much wider area, while eroded materials often end in areas not suitable for crop growth (river beds, estuaries, etc.) or water bodies (wetlands, reservoirs, ocean). The weight of GHG emissions from eroded area could have been much larger, and depositional zone may have led to additional CH_4_ emissions. This calls for systematic investigation in the future study.

## Methods

### Study area

The study was conducted in an on-going long-term experiment at Waterman Farm of the Ohio State University, Columbus, OH, USA (N + 40° 1′ 5.52″ E −83° 2′ 29.72″). The experiment was initiated in 1997 on the Crosby soil series (deep, fine, mixed, active, mesic, Aeric Epiaqualf). The deep soil developed on nearly level topography (0% to 2% slopes) is of silt loam texture, poorly drained and derived from glacial till. The mean annual rainfall is 1016 mm and the mean annual air temperature is 11 °C ^8^.

The experiments were designed in a split-plot arrangement with completely randomized blocks. Three TSD levels were carried out as main plots and two amendment types as subplots. The 18 × 9 m main plots were subdivided into 6 × 4.5 m subplots, with three replications for each treatment combination. The main plots were separated by 2.7 m long border strips^8,38^. Three TSD levels created once at the beginning of the experiment to simulated soil erosion- deposition process, which were: (1) topsoil removal (eroded site) created by physically remove of 20 cm topsoil with a landscape loader; (2) undisturbed control (uneroded site); and (3) topsoil addition (depositional site) achieved by deposit of 20 cm topsoil on soil surface. Two amendments were applied in this study: N-fertilizer and organic manure. For the plots receiving N-fertilizer, 150 kg N ha^−1^ urea-ammonium nitrate (28% N) was side-banded on the soil surface at the 3^rd^ to 4^th^ leaf stage of corn growth. For the plots receiving organic manure, dry matter compost (20 Mg ha^−1^) was uniformly top dressed during April each year.

### CO_2_, N_2_O and CH_4_ flux measurements

Soil-air samples for the assessment of CO_2_, N_2_O and CH_4_ fluxes were collected using the static chamber method^42^. Gas chambers were made of polyvinyl chloride (PVC) pipes of 15 cm diameter and 30 cm length. The top lid was made of a PVC cap, and the lower end was trimmed to be inserted into the soil. A machine-trimmed PVC trough was coupled around the outer ring of the pipe, approximately 5 cm from the top. The PVC cap was equipped with a sampling port and a rubber septum on the top, and the cap bottom could be fitted into the trough when the cap was in place^42,43^.

The chambers were inserted 10 cm deep into the ground at each sampling point, with three replications for each treatment. Chambers were installed 1 month before gas sampling, and the chambers remained in place with the cap opened during the entire growing season, except for temporary removal during seeding or fertilizer application. Chambers were reinstalled in the same place immediately after the fertilizer and seeding operations were completed^34^.

When sampling, closed the chamber lid, taken approximately 10 cm^3^ soil-air samples from each chamber headspace at 0 and 30 minutes, and transferred it to crimp sealed pre-evacuated 10 ml vials fitted with butyl rubber septa. The vials were evacuated to a pressure of −172 kPa and prepared 1 day before sampling. Soil-air samples were obtained between 11 AM and 2 PM when fluxes were expected to be maximal^43^ biweekly during the entire growing season. Three replications were taken for each treatment.

The CO_2_ and CH_4_ in soil-air samples were analyzed using a GC-2014 gas chromatograph (GC; Shimadzu, Kyoto, Japan) equipped with a thermal conductivity detector for CO_2_, and a flame ionization detector for CH_4_. N_2_O was analyzed on a GC fitted with a ^63^Ni electron capture detector^34^.

### Soil temperature and moisture measurements

Soil samples for measurements of soil temperature and soil moisture content were collected biweekly in conjunction with soil-air sampling^18^. Soil temperatures at 10 cm soil depth were monitored by using a digital thermometer near each chamber simultaneously with gas sampling. Gravimetric soil moisture content was also determined by collecting soil samples close to the chambers at 0–10 cm depth.

### Analysis of soil properties

Bulk and intact core samples were obtained separately in June 2012 to measure soil properties. Intact core soil samples for bulk density analysis were collected at 0–40 cm depth (10 cm intervals) using a manually-driven core sampler with diameter and height both 5 cm. Gravimetric soil moisture content (SMC) was measured by drying a portion of trimmed core samples at 105 °C for 24 h^19^. Wet bulk density was computed as the ratio of soil wet weight to core volume, and soil bulk density was calculated from the wet bulk density and soil moisture content, *ρ*_*b*_ = *ρ*_*b*_′/(1 + *w*), where *ρ*_*b*_ is soil bulk density, *ρ*_*b*_′ is soil wet bulk density, and *w* is the gravimetric moisture content. Total porosity was calculated from the equation^44^: *f* = 1 − (*ρ*_*b*_*′*/*ρ*_*s*_), where *ρ*_*s*_ is the soil particle density and is estimated at 2.65 g cm^−3^. Bulk soil samples were air-dried at room temperature, ground with a wooden hammer, and sieved through a 2 mm sieve before physical and chemical analysis^38^. Soil total C and N contents were analyzed by the dry combustion method using a vario Max CN analyzer (Elementar, Hanau, Germany)^18^. The SOC was assumed to be equal to the total C as inorganic C concentration was negligible with the soil pH was below 7^38^.

### Crop yield

Corn (*Zea mays* L.) was grown from about mid-May to October in 2012 without any major disturbances, and no tillage operation was performed. Corn plants from the center two rows of each plot were hand harvested. Crop residue after the harvest was left on the soil surface.

Corn ears were separated from the stover and weighed. Corn ears were shelled, and grains were weighed separately from other parts of the ear after air drying. Subsamples of grain were weighed and then oven-dried at 60 °C for 48 h to determine the water content^38^. Grain yields are reported in Mg ha^–1^ at 12% moisture content.

### Data calculations and statistical analysis

Daily gas fluxes (*q*) (in units of g CO_2_–C m^−2^ d^−1^ or mg N_2_O − N m^−2^ d^−1^ or mg CH_4_ − C m^−2^ d^−1^) were computed using Eq. (1)^34,42^:

1 $${\rm{q}}=(\frac{{\rm{\Delta }}C{O}_{2}-C\,\,or\,{\rm{\Delta }}{N}_{2}O-N\,or\,C{H}_{4}-C}{{\rm{\Delta }}t})(\frac{V}{A})k$$

where (ΔCO_2_–C or ΔN_2_O–N or ΔCH_4_–C)/Δt is the rate of gaseous accumulation inside the chamber (e.g., g CO_2_–C m^−3^ air min^−1^, mg N_2_O–N m^−3^ air min^−1^ or mg CH_4_–C m^−3^ air min^−1^), *V* is the volume of the chamber (m^3^), *A* is the surface area coverd by the chamber (m^2^), and *k* is a time conversion factor (1440 min day^−1^).

Seasonal gas emissions were estimated as the cumulative amount of CO_2_, N_2_O or CH_4_ emitted during the growing season. Recalculations were made through linear interpolation of two neighboring measured fluxes and numerical integration over time, per Eq. (2)^45^:

2 $$C{O}_{2}-C\,{\rm{or}}\,{N}_{2}O-N\,or\,C{H}_{4}-C=\sum _{i}^{n}[({x}_{i}+{x}_{i+1})\times \frac{N}{2}]+\cdots +[({x}_{n-1}+{x}_{n})\times \frac{N}{2}]$$

where *i* = date of the first measurement taken of CO_2_ or N_2_O or CH_4_ rate, *n* = date of the last measurement taken of CO_2_, N_2_O or CH_4_ rate; *x* = CO_2_ rate (g m^2^ d^−1^), N_2_O rate (mg m^2^ d^−1^) or CH_4_ rate (mg m^2^ d^−1^); and *N* = number of days between the two consecutive CO_2_, N_2_O or CH_4_ rate measurements.

The CO_2_ equivalents were computed by Eq. (3), converting the cumulative GHG emission to GWP, using factors of 310 and 21 for N_2_O and CH_4_, respectively^34^:

3 $${\rm{GWP}}={\rm{C}}{{\rm{H}}}_{{\rm{4}}}\times 21+{{\rm{N}}}_{{\rm{2}}}{\rm{O}}\times 310+{\rm{C}}{{\rm{O}}}_{{\rm{2}}}({\rm{M}}{\rm{g}}\,{\rm{C}}{{\rm{O}}}_{{\rm{2}}}{\rm{E}}{\rm{q}}\,{\rm{h}}{{\rm{a}}}^{-1}{\rm{g}}{\rm{r}}{\rm{o}}{\rm{w}}{\rm{i}}{\rm{n}}{\rm{g}}\,{\rm{s}}{\rm{e}}{\rm{a}}{\rm{s}}{\rm{o}}{{\rm{n}}}^{-1})$$

The greenhouse gas intensity (GHGI) was calculated by dividing GWP by crop yield using Eq. (4)^46^:

4 $${\rm{G}}{\rm{H}}{\rm{G}}{\rm{I}}={\rm{G}}{\rm{W}}{\rm{P}}/{\rm{g}}{\rm{r}}{\rm{a}}{\rm{i}}{\rm{n}}\,{\rm{y}}{\rm{i}}{\rm{e}}{\rm{l}}{\rm{d}}({\rm{M}}{\rm{g}}\,{{\rm{C}}{\rm{O}}}_{2}{\rm{E}}{\rm{q}}\,{\rm{M}}{\rm{g}}\,{\rm{g}}{\rm{r}}{\rm{a}}{\rm{i}}{\rm{n}}\,{\rm{y}}{\rm{i}}{\rm{e}}{\rm{l}}{\rm{d}}\,{\rm{g}}{\rm{r}}{\rm{o}}{\rm{w}}{\rm{i}}{\rm{n}}{\rm{g}}\,{{\rm{s}}{\rm{e}}{\rm{a}}{\rm{s}}{\rm{o}}{\rm{n}}}^{-1})$$

Statistical analysis was performed using the analysis of variance (ANOVA) procedure available in SAS 8.01 for Windows (1999–2000, SAS Institute Inc., Cary, NC, USA). Mean and interactive effects of treatments were separated using the F-protected least significant difference test. The probability level (*P*) chosen to designate significance was ≤0.05. Correlation and regression analyses were performed on selected variables at *P* ≤ 0.1 using the same package.

### Data availability statement

The datasets generated during the current study are available from the corresponding author on reasonable request.
